# Characterization of the complete plastome of *Aster pekinensis* (Asteraceae), a perennial herb

**DOI:** 10.1080/23802359.2021.1899081

**Published:** 2021-03-19

**Authors:** Xin Zhang, Pei-Pei Jiang, Shou-Jin Fan

**Affiliations:** Shandong Provincial Key Laboratory of Plant Stress Research, College of Life Sciences, Shandong Normal University, Ji’nan, China

**Keywords:** *Aster pekinensis*, plastome, phylogeny

## Abstract

*Aster pekinensis* is a perennial herb that distributes widely in China, Korea, and Eeastern Russia. The complete plastome of *A. pekinensis* is reported here. It is a circular molecular of 152,815 bp in length and consists of a large single-copy region (LSC: 84,530 bp), a small single-copy region (SSC: 18,219 bp), and two inverted repeats (IR: 25,033 bp) regions. GC content is 37.3%. This plastome encodes 113 unique genes, including 79 protein-coding genes, 30 tRNAs, and 4 rRNAs. Phylogenomic analysis of 17 plastomes within *Aster* and closely related genera revealed that *A. pekinensis* was sister to the clade comprising *A. flaccidus* and *A. altaicus*.

*Aster* is a genus of the family Asteraceae. This genus has 152 species, 80% of which are distributed in China. *A. pekinensis* is a widespread perennial herb growing on forest margins, thickets, mountain slopes, riverbanks, and roadsides (Chen et al. 2011). Due to its good quality and palatability, it can be used as a kind of forage grass (Yang and Li 2003). Based on combined sequences of ITS, ETS, and *trnL-F*, the recent phylogenetic tree demonstrated that *A. pekinensis* was sister to *A. indicus* (Zhang et al. 2019). In this study, we reported the complete plastome of *A. pekinensis*, which will be helpful for species identification and the phylogenetic analysis of the genus *Aster*.

Silica-dried leaves of *A. pekinensis* were collected from Zibo, Shandong, China (118°6′10.01″E, 36°23′32.16″N). Voucher specimen (No.198-2) was deposited at School of Life Sciences, Shandong Normal University. Total genomic DNA was isolated using a modified CTAB-based protocol (Wang et al. 2013). DNA library preparation and sequencing were conducted by Illumina Novaseq platform at Novogene (Beijing, China). After obtaining sequencing data, we used Organelle Genome Assembler (OGA, https://github.com/quxiaojian/OGA) to assemble plastome (Qu, Fan, et al. 2019). Annotation was performed with Plastid Genome Annotator (PGA, https://github.com/quxiaojian/PGA) (Qu, Moore, et al. 2019). Referring to previous published studies (Qu 2019; Wang et al. 2019), we used Geneious v9.1.4 to do manual correction (https://www.geneious.com). All 79 protein-coding genes were selected to construct the maximum likelihood (ML) tree by RAxML v8.2.10 (Stamatakis 2014), using 1000 bootstrap replicates with GTRCAT model after alignment using MAFFT v7.313 (Katoh and Standley 2013).

The complete plastome of *A. pekinensis* (GenBank accession number: MW255593) is a circular molecular of 152,815 bp in length. It consists of a large single-copy region (LSC: 84,530 bp), a small single-copy region (SSC: 18,219 bp), and two inverted repeats regions (IRs: 25,033 bp). GC content is 37.3%. This plastome encodes 113 unique genes, including 79 protein-coding genes, 30 tRNAs, and 4 rRNAs. There are genes with two copies, including *rps12*, *ycf2*, *rrn23*, *ndhB*, *rpl2*, *rrn16*, *trnA-UGC*, *trnI-GAU*, *rps7*, *rpl23*, *rrn5*, *rrn4.5*, *trnL-CAA*, *trnI-CAU*, *trnR-ACG*, *trnV-GAC*, and *trnN-GUU* gene. Phylogenomic analysis of 17 plastomes within *Aster* and its related genera revealed that *A. pekinensis* was sister to the clade comprising *A. flaccidus* and *A. altaicus* (Figure 1).

**Figure 1.**
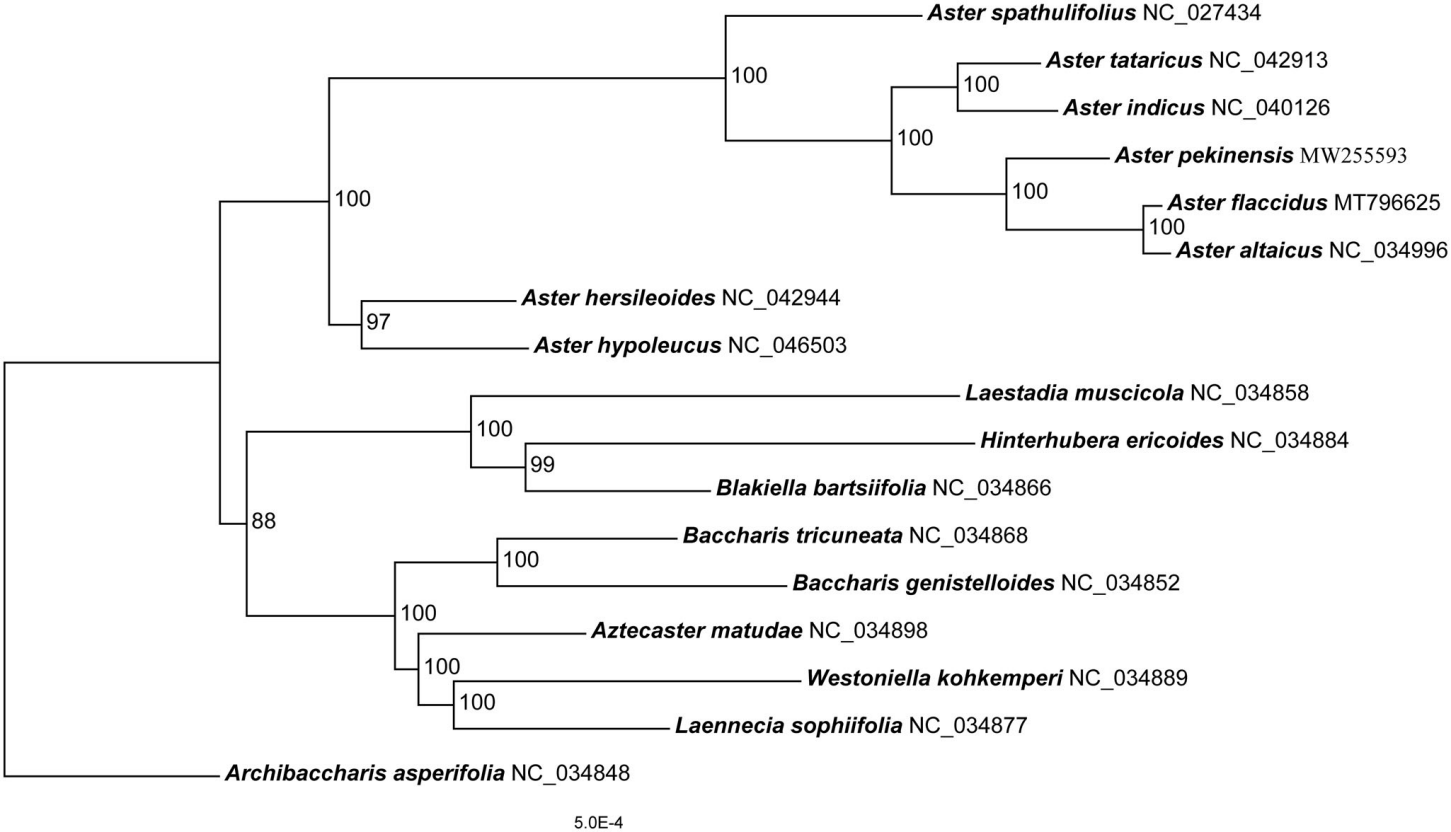
A maximum likelihood (ML) phylogenetic tree based on 17 Asteraceae species is shown. Bootstrap support values are shown as numbers next to branches.

## Data avaliability statement

The data that support the findings of this study are openly available in GenBank of NCBI at https://www.ncbi.nlm.nih.gov, reference number MW255593. The associated BioProject, SRA, and Bio-Sample numbers are PRJNA679197, SUB8572223, and SAMN16825934, respectively.
